# *Escherichia coli* Culture Filtrate Enhances the Growth of *Gemmata* spp.

**DOI:** 10.3389/fmicb.2019.02552

**Published:** 2019-11-06

**Authors:** Odilon D. Kaboré, Rita Aghnatios, Sylvain Godreuil, Michel Drancourt

**Affiliations:** ^1^IHU Méditerranée Infection, Marseille, France; ^2^Aix-Marseille Université, IRD, MEPHI, IHU Méditerranée Infection, Marseille, France; ^3^Département de Bactériologie-Virologie, Centre Hospitalier Universitaire de Montpellier, Montpellier, France

**Keywords:** Planctomycetes, *Gemmata obscuriglobus*, *Gemmata massiliana*, *E. coli* culture filtrate, iron, culture

## Abstract

**Background:**

Planctomycetes bacteria are known to be difficult to isolate, we hypothesized this may be due to missing iron compounds known to be important for other bacteria. We tested the growth-enhancement effect of complementing two standard media with *Escherichia coli* culture filtrate on two cultured strains of *Gemmata* spp. Also, the acquisition of iron by *Gemmata* spp. was evaluated by measuring various molecules involved in iron metabolism.

**Materials and Methods:**

*Gemmata obscuriglobus* and *Gemmata massiliana* were cultured in Caulobacter and Staley’s medium supplemented or not with *E. coli* culture filtrate, likely containing siderophores and extracellular ferrireductases. We performed iron metabolism studies with FeSO_4_, FeCl_3_ and deferoxamine in the cultures with the *E. coli* filtrate and the controls.

**Results and Discussion:**

The numbers of *G. obscuriglobus* and *G. massiliana* colonies on Caulobacter medium or Staley’s medium supplemented with *E. coli* culture filtrate were significantly higher than those on the standard medium (*p* < 0.0001). Agar plate assays revealed that the *Gemmata* colonies near *E. coli* colonies were larger than the more distant colonies, suggesting the diffusion of unknown growth promoting molecules. The inclusion of 10^–4^ to 10^–3^ M FeSO_4_ resulted in rapid *Gemmata* spp. growth (4–5 days compared with 8–9 days for the controls), suggesting that both species can utilize FeSO_4_ to boost their growth. In contrast, deferoxamine slowed down and prevented *Gemmata* spp. growth. Further studies revealed that the complementation of Caulobacter medium with *E. coli* culture filtrate and 10^–4^ M FeSO_4_ exerted a significant growth-enhancement effect compared with that obtained with Caulobacter medium supplemented with *E. coli* culture filtrate alone (*p* < 0.0122). Moreover, the intracellular iron concentrations in *G. obscuriglobus* and *G. massiliana* cultures in iron-depleted broth supplemented with the *E. coli* filtrate were 0.63 ± 0.16 and 0.78 ± 0.12 μmol/L, respectively, whereas concentrations of 1.72 ± 0.13 and 1.56± 0.11 μmol/L were found in the *G. obscuriglobus* and *G. massiliana* cultures grown in broth supplemented with the *E. coli* filtrate and FeSO_4_. The data reported here indicated that both *E. coli* culture filtrate and FeSO_4_ act as growth factors for *Gemmata* spp. via a potentiation mechanism.

## Background

Bacteria of the genus *Gemmata* belong to the superphylum *Planctomycetes–Verrucomicrobia–Chlamydia* (PVC) and the phylum Planctomycetes (Wagner and Horn, 2006). Similarly to other members of Planctomycetes, *Gemmata* bacteria constitute one of the phylogenetically distinct major groups with increasing relevance to research in microbial ecology, molecular evolution, cell biology, and most recently, clinical microbiology (Fuerst, 2004; Drancourt et al., 2014; Aghnatios and Drancourt, 2016; van Niftrik and Devos, 2017). Indeed, some biologists now claim that *Gemmata* bacteria are nucleus−bearing prokaryotes but are considered evolutionary intermediates in the transition from prokaryote to eukaryote due to their amazingly complex cellular architectures that are typical of eukaryotes, such as those associated with cytosolic compartmentalization (Santarella-Mellwig et al., 2013; Sagulenko et al., 2014), sterol synthesis (Pearson et al., 2003; Gudde et al., 2019) and endocytosis-like macromolecular uptake (Lonhienne et al., 2010; Boedeker et al., 2017). These species form a remarkable gram-negative-staining group of bacteria that exhibit characteristic bud production, and a division process independent of FtsZ via budding-mediated polar fission, which is different from that of ordinary bacteria, where FtsZ is the main molecule involved in cell division (Fuerst, 2004; Bernander and Ettema, 2010). Both *G. obscuriglobus* and *G. massiliana* are slow-growing, fastidious organisms, and *G. obscuriglobus* exhibits a 13-h doubling time (Lee et al., 2009). *Gemmata* bacteria require highly specific culture media and long incubation times (Schlesner, 1994; Winkelmann and Harder, 2009; Lage and Bondoso, 2012; Mishek et al., 2018). We recently found some *Gemmata*-like sequences in blood collected from two patients with febrile aplastic neutropenia and leukemia, although we failed to isolate any Planctomycetes from these blood samples (Drancourt et al., 2014). Accordingly, conventional automated microbial detection of blood culture systems is not appropriate for the detection of these type of bacteria (undetected) and is less sensitive than the culture of mock-infected blood on Caulobacter agar (Christen et al., 2018). Nevertheless, the resistance of these bacteria to most of the routinely used antibiotics (Cayrou et al., 2010a; Godinho et al., 2019) and their recently demonstrated association with humans (Cayrou et al., 2013; Drancourt et al., 2014) support the potential behavior of *Gemmata* organisms as opportunistic pathogens, and this hypothesis warrants further investigations (Aghnatios and Drancourt, 2016).

The culture-based isolation of microbial pathogens remains the gold standard in diagnostic microbiological laboratories, but it has been reported that the lack of complex factors/conditions in these laboratories contributes to the inability to isolate some fastidious bacterial species. Accordingly, the provision of environmental and nutritional conditions similar to those existing in the natural habitat where yet-uncultured/refractory bacteria can be detected might be an option for their potential isolation and culture (Kaeberlein et al., 2002; Vartoukian et al., 2010). Some yet uncultured planctomycetes, such as *Planctomycetes bekefii*, possess stalks encrusted with iron oxide deposits (Schmidt et al., 1981), but the associated mechanism (active oxidation or passive deposition) has not been determined, and these findings suggest an important role for iron in these organisms. Our preliminary genome analysis of *Gemmata obscuriglobus* [UQM 2246 (GenBank: NZ_ABGO00000000.1)] and *G. massiliana* [GenBank: CBXA000000000.1], which are the only cultured representatives of the Planctomycetes genus *Gemmata* that have been formally described (Franzmann and Skerman, 1984; Aghnatios et al., 2015) using the Rapid Annotation Subsystem Technology server (Meyer et al., 2008), revealed that these bacteria do not contain molecules involved in the iron acquisition pathway, which might partially explain their notable fastidiousness when grown on culture media. We thus hypothesized that supplementation of the standard culture media (Caulobacter and Stanley) for *Gemmata* spp. with *Escherichia coli* culture filtrate and iron (“ecological” medium) could enhance their growth and isolation in clinical microbiology laboratories.

## Materials and Methods

### Bacterial Strains

*Gemmata obscuriglobus* DSM 5831^T^ and *G. massiliana* DSM 26013^T^ (CSUR P189^T^) were obtained from the Collection de Souches de l’Unité des Rickettsies (Marseille, France) and the German Collection of Microorganisms and Cell Cultures (Braunschweig, Germany). Both species were subcultured on Caulobacter medium DSMZ 595 or Staley’s maintenance medium DSMZ 629 prepared as described on the website^1^ The bacteria were grown through aerobic incubation on these solid media at 30°C for 7 to 14 days. The colonies was identified by matrix-assisted laser desorption/ionization time-of-flight mass spectrometry (MALDI-TOF-MS) analysis as previously described (Cayrou et al., 2010b).

### *Escherichia coli* Culture Filtrate Preparation

*Escherichia coli* strain CIP 7624 (Collection de l’Institut Pasteur, Paris, France) was initially cultured on blood agar (BioMérieux, Marcy-l’Étoile, France) for 24 h at 37°C and identified by MALDI-TOF-MS as previously described (Seng et al., 2009). The bacterial cell counts were calibrated to 10^12^ colony forming units (CFUs)/mL using Kovas slide 10 (Hycor Biomedical, Germany) and microscopic examination. One milliliter of this suspension was then subcultured in 75-cm^2^ culture flasks containing 49 mL of autoclaved GLD medium (1 g of glucose, 1.4 g of peptone, 0.3 g of NaCl, 20 mL of Hutner’s salt (DSMZ 590), 10 mL of Staley vitamins (DSMZ 600, added after filter-sterilized) and 970 mL of distilled water) and incubated aerobically with shaking at 250 rpm for 2 days at 30°C to elicit the release of *E. coli* siderophores in a low-iron environment (Miethke and Marahiel, 2007). Sonication was performed to increase the release of *E-coli* siderophores, as previously described (Kwon and Jewett, 2015). Briefly, the cells were transferred to 1.5-mL microtubes and sonicated in a water bath sonicator (Bransonic^®^ Ultrasonic Cleaner Model 5510R-MT, Branson Ultrasonic Corporation) at ∼20°C, a frequency of 20 kHz and an amplitude of 50% for 1 × 2 h. Subsequently, the sonication broth was filtered through a 0.2 μm filter (Sigma-Aldrich, Saint-Quentin-Fallavier, France) to obtain the *E. coli* filtrate named solution A. Solution B was prepared in the same manner as solution A with the exception that the GLD medium was supplemented with 10^–4^ M ferrous sulfate heptahydrate (Sigma-Aldrich) and the culture was incubated for 3 days and then filtered. Solution B was prepared with the aim of inducing the production of extracellular iron reductase by *E. coli* in an iron-rich environment. As a negative control, autoclaved noninoculated GLD medium was manipulated under the same conditions as the inoculated culture flasks. Finally, 10 μL of solution A, solution B and the control GLD medium were seeded in blood, Staley’s and Caulobacter solid agar to ensure sterility.

### Culture of *Gemmata* spp. on Caulobacter and Staley’s Liquid Media With *E. coli* Filtrate

*Gemmata obscuriglobus* and *G. massiliana* were cultured independently in five replicates in a final volume of Caulobacter liquid medium of 15 mL. In detail, five tubes contained 9 mL of Caulobacter liquid medium supplemented with 5 mL of *E. coli* filtrate (2.5 mL of solution A + 2.5 mL of solution B), and five tubes contained 9 mL of Caulobacter liquid medium supplemented with 5 mL of GLD medium (negative controls). Each tube (five test tubes and five control tubes) was inoculated with 1 mL of 3.10^2^ CFUs/mL suspended in sterile distilled water (Bio-Rad Laboratories, Hercules, CA, United States). Moreover, two test tubes and two control tubes were inoculated with 1 mL of sterile distilled water (noninoculated tubes) and manipulated in parallel to the negative control tubes. The preparations were then incubated at 30°C in an aerobic atmosphere for 7 days. At days 1, 2, 3, 4, and 7 postinoculation, each tube was shaken, and 1 mL of the broth was removed to obtain serial dilutions of 1, 1/10, 1/100, 1/1000, and 1/10000 in sterile distilled water for culture-based microbial enumerations. The CFUs were enumerated on 100-mm Petri dishes containing Caulobacter solid agar, and the colonies were counted using scanning software (ImageJ, Interscience, Saint-Nom-la-Bretèche, France). The means and standard errors were calculated at each time point (five replicates, *n* = 5). All experiments were reproduced independently with *G. obscuriglobus* in Staley’s liquid medium, and these were performed in parallel to those conducted with *G. massiliana*.

### Culture of *Gemmata* spp. Under Iron-Repleted and Iron-Depleted Conditions in the Presence or Absence of *E. coli* Culture Filtrate, FeSO_4_, FeCl_3_ and Deferoxamine

Five experiments with Caulobacter liquid medium (*Gemmata* spp. grown in standard iron-free medium compared with Staley’s medium, which contains FeSO_4_) were performed independently. The iron metabolism in assay tubes containing Caulobacter liquid medium in the presence of *E. coli* culture filtrate under iron-repleted, iron-depleted and control conditions was studied. Ferrous iron heptahydrate (FeSO_4_●7H_2_O, Sigma Aldrich), ferric chloride (FeCl_3_, Sigma Aldrich) and deferoxamine mesylate (Desferal^®^, Novartis, Rueil-Malmaison, France) were used to probe iron assimilation. Each of these components was added to a final concentration of 10^–4^ M in a final volume of 15 mL (appropriately low concentrations of *E. coli* filtrate (5%), FeSO_4_ (0.2 M), FeCl_3_ (0.2 M) and deferoxamine (100 mg/mL), as determined by serial dilution of 0.2 M to 10^–4^ M; see Table 1 for the oxidation-reduction potential (ORP) and pH at 25°C obtained with all initial solutions used). In detail, the first tube contained 10^–4^ M FeSO_4_, the second tube contained 10^–4^ M FeCl_3_, the third tube contained 10^–4^ M deferoxamine, the fourth tube contained 10^–4^ M FeSO_4_ + 10^–4^ M deferoxamine, the fifth tube contained 10^–4^ M FeCl_3_ + 10^–4^ M deferoxamine dissolved in Caulobacter liquid medium, and the last tube contained only Caulobacter liquid medium. In parallel, six other tubes contained 9 mL of Caulobacter liquid medium supplemented with 5 mL of *E. coli* culture filtrate (2.5 mL of solution A + 2.5 mL of solution B), and each of these components was added to a final concentration of 10^–4^ M in a final volume of 15 mL, as described above. Subsequently, the 12 tubes were inoculated with 1 mL of 3 × 10^2^ CFUs/mL suspended in Caulobacter liquid medium and incubated aerobically at 30°C for 7 days. One noninoculated (negative control) tube for each of the 12 tubes was manipulated in parallel. At days 1, 2, 3, 4, and 7 postinoculation, each tube was shaken, and 1 mL was removed to obtain serial dilutions of 1, 1/10, 1/100, 1/1000, and 1/10000 in distilled sterile water for culture-based CFU enumerations on Caulobacter solid agar. In addition, daily measurements of the ORP and pH at 25°C (accumet^®^ AE150, Fisher Scientific) of each liquid medium were performed in parallel. Moreover, 2 × 50 μL of each liquid medium was adsorbed on blotting paper and deposited on solid medium in parallel to observe the growth time around the blotting paper. Furthermore, for each tube, 100 mm Petri dishes containing solid agar were prepared in parallel to monitor the growth on solid media (colony features, color and growth time in the presence or absence of *E. coli* filtrate), and these contained all the above-mentioned components at the same final concentrations. The Petri dishes prepared to contain *E. coli* filtrate were supplemented with 500 μL of solution A and 500 μL of solution B and dried at room temperature for 30 min in a laminar flow cabinet. The noninoculated (negative control) tubes and Petri dishes were manipulated in parallel. The bacteria were then counted using scanning software. *G. massiliana* and *G. obscuriglobus* were cultured independently in the same manner. The amount of intracellular iron was quantified after incubation for 1 and 7 days. Ten microliters of each liquid culture were inoculated on Caulobacter solid medium and Caulobacter solid medium complemented with each component as described above to monitor the bacterial features, survival and contamination. After incubation for 7 days, the liquid medium was centrifuged at 1.1 *g* for 5 min, and the pellet was washed three times with 10^–4^ M deferoxamine. The concentration of iron was measured using a colorimetric ferrozine method as previously described (Riemer et al., 2004). Briefly, 200 μL of 50 mM NaOH, 200 μL of 10 mM HCl and 200 μL of iron-releasing solution were added to the specimens, and the mixtures were incubated for 2 h at 60°C. All the solutions were then filtered through a 0.2-μm filter, and the iron concentration in a 350 μL aliquot was measured using an Iron 2 Cobas kit (Cobas, Meylan, France).

**TABLE 1:** Ph and ORP measured of solution used to study iron acquisition.

	**PH (25°C)**	**ORP (mv, 25°C)**	**Concentration**
FeCl_3_	3.13	233	0.2 M
FeSO_4_	3.6	202	0.2 M
Desf	3.77	61.3	100 mg/mL
Caulobacter	7.24	–13.7	–
Filtrate	7.46	–29.4	5%

### *Gemmata* spp. Growth on Petri Dishes in the Presence of a Panel of Molecules Involved in Iron Metabolism Absorbed in Sterile Blotting Paper

Live *E. coli* (soaked in sterile blotting paper, used as a helper strain) was cultured in close proximity to *G. obscuriglobus* and *G. massiliana* to assess its ability to promote the growth of these *Gemmata* bacteria. The growth of *Gemmata* spp. in the presence of *E. coli* filtrate (prepared in simple Caulobacter liquid medium) was then assessed through plate assays as previously described by D’Onofrio et al. (2010). Briefly, 2 × 50 μL of each solution containing various molecules involved in iron metabolism, namely, FeSO_4_, FeCl_3_, FeSO_4_ and deferoxamine at concentrations of 0.2, 10^–1^, 10^–2^, 10^–3^, and 10^–4^ M, with and without *E. coli* filtrate was triturated and adsorbed on blotting paper to study the influence of these components on *Gemmata* growth in Caulobacter medium through plate assays.

## Results and Discussion

To the best of our knowledge, no member of Planctomycetes has been isolated from clinical samples, even though Planctomycetes bacteria have recently been detected in aplastic patients by PCR (Drancourt et al., 2014). This study aimed to develop an optimal medium for the culture and recovery of fastidious *Gemmata* bacteria in our laboratory using an “ecological” medium. Hence, this study was performed from a translational perspective for environmental/clinical microbiologists, and the results should not be translated to mechanistic studies conducted in clinical microbiology laboratories aiming to describe the iron metabolism of fastidious *Gemmata*.

**FIGURE 1:**
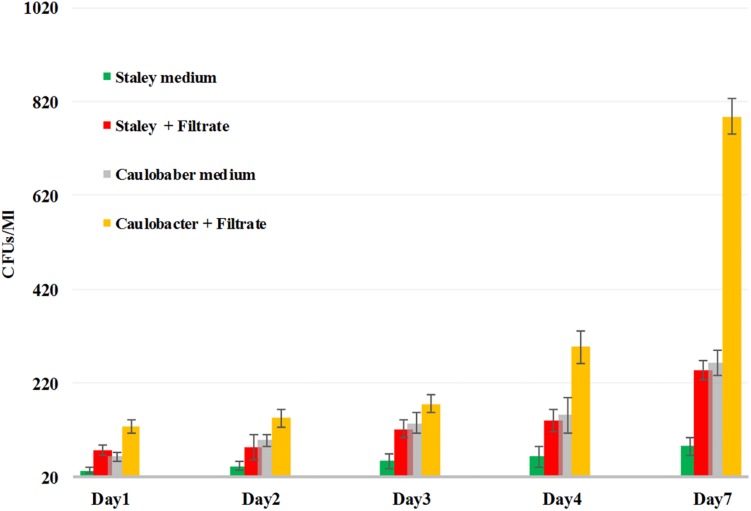
*G. obscuriglobus* growth in standard Caulobacter medium (gray bar), standard Staley medium (green bar), Caulobacter medium supplemented with *E. coli* filtrate (yellow bar) and Staley medium supplemented with *E. coli* filtrate (red bar). The number of *G. obscuriglobus* colonies per milliliter (*Y* axis) on solid agar medium was monitored over a 7-day period (*X* axis).

**FIGURE 2:**
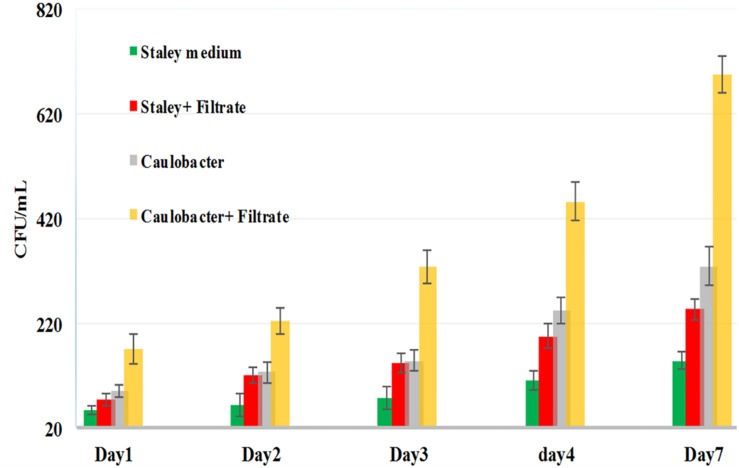
*G. massiliana* growth in standard Caulobacter medium (gray bar), standard Staley medium (green bar), Caulobacter medium supplemented with *E. coli* filtrate (yellow bar) and Staley medium supplemented with *E. coli* filtrate (red bar). The number of *G. massiliana* colonies per milliliter (*Y* axis) on solid agar medium was monitored over a 7-day period (*X* axis).

**FIGURE 3:**
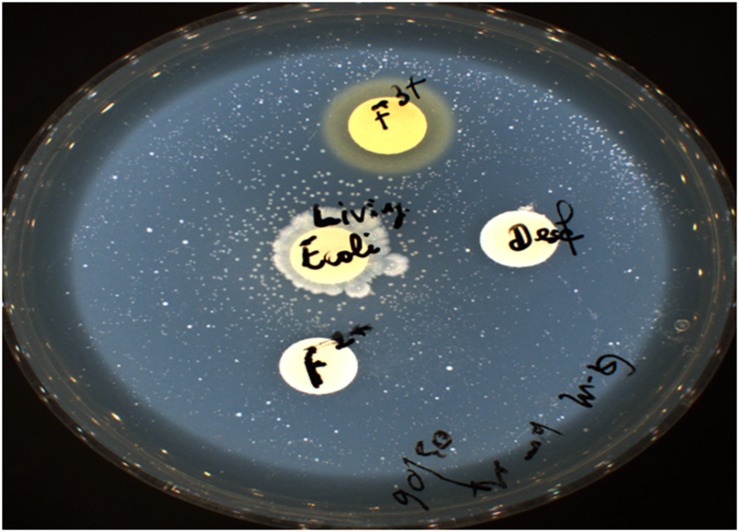
Live *E. coli* promotes the growth of *Gemmata massiliana*. Ferric and ferrous iron at 0.2 M are toxic to *Gemmata*, and 10^–4^ M deferoxamine prevents bacterial growth.

**FIGURE 4:**
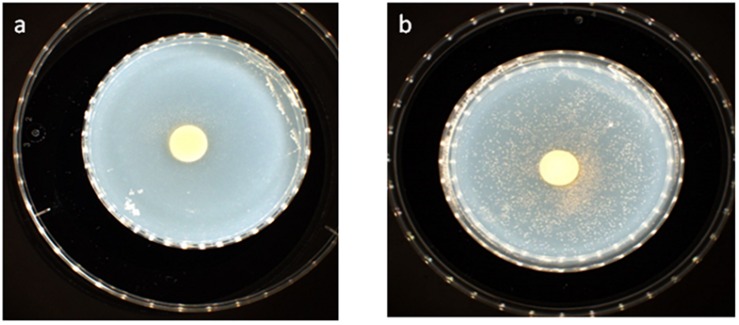
*Gemmata massiliana* showed improved growth in blotting paper impregnated with 10^–3^ M FeSO_4_ (50 mL) nitrocellulose disks at day 4 **(a)**, whereas small colonies did not begin to appear until days 8 and 9 in more distant areas in the disks **(b)**.

**FIGURE 5:**
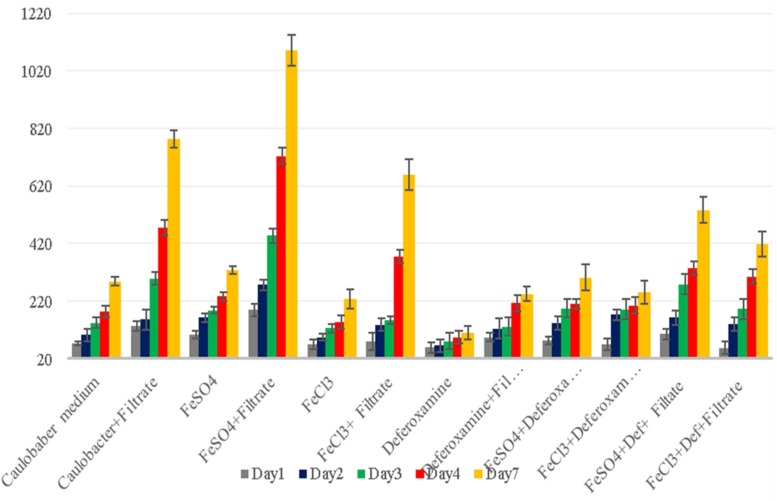
*G. obscuriglobus* growth in standard Caulobacter medium supplemented with *E. coli* filtrate, FeSO_4_, FeCl_3_, and deferoxamine and control medium. The number of *G. obscuriglobus* colonies per milliliter (*Y* axis) on solid agar medium was monitored over a 7-day period (*X* axis).

**FIGURE 6:**
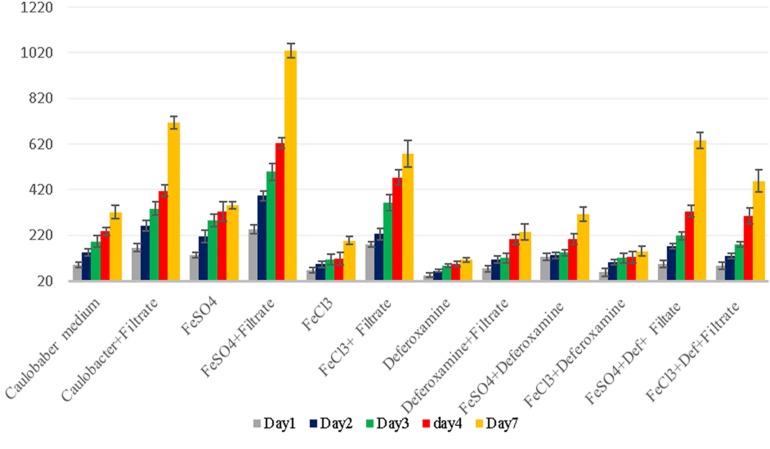
*G. massiliana* growth in standard Caulobacter medium supplemented with *E. coli* filtrate, FeSO_4_, FeCl_3_, and deferoxamine and control medium. The number of *G. massiliana* colonies per milliliter (*Y* axis) on solid agar medium was monitored over a 7-day period (*X* axis).

Thus, we reasoned that the enhancement in the growth of *Gemmata obscuriglobus* and *Gemmata massiliana* obtained by supplementation with filtrates of *E. coli* cultures and iron at low concentrations (5% filtrates and 10^–4^ M FeSO_4_) reduce the doubling time of these fastidious bacteria potentially via a potentiation mechanism. Indeed, our observations revealed that although the noninoculated (negative) controls remained sterile throughout the experiments, the number of *G. obscuriglobus* colonies on Caulobacter medium supplemented with *E. coli* filtrate (126 ± 13 colonies on day 1 and 787 ± 38 colonies on day 7) was significantly higher than that on the standard medium (62 ± 10 colonies on day 1 and 261 ± 27 colonies on day 7) (*p* < 0.0001). Similarly, the number of *G. obscuriglobus* colonies on Staley’s medium supplemented with *E. coli* filtrate (75 ± 11 colonies on day 1 and 247 ± 20 colonies on day 7) was significantly higher than that on the standard medium (32 ± 6 colonies on day 1 and 82 ± 18 colonies on day 7) (*p* < 0.0001) (Figure 1). For *G. massiliana*, the number of colonies on the medium supplemented with *E. coli* filtrate (Caulobacter medium, 170 ± 29 colonies on day 1 and 694 ± 35 colonies on day 7; Staley medium, 74 ± 12 colonies on day 1 and 246 ± 21 colonies on day 7) was significantly higher than that on the standard medium (Caulobacter medium, 89 ± 11 colonies on day 1 and 329 ± 37 colonies on day 7, *p* < 0.0001; Staley medium, 54 ± 8 colonies on day 1 and 148 ± 17 colonies on day 7, *p* < 0.0001) (Figure 2). Altogether, a significantly higher number of *Gemmata* spp. colonies was obtained after enrichment of the reference culture medium with *E. coli* filtrate (*p* < 0.0001). Surprisingly, the growth of *Gemmata s*pp. on Caulobacter medium supplemented with *E. coli* filtrate was improved compared with that on Staley’s medium supplemented with *E. coli* culture filtrate (Figures 1, 2), even though Staley’s medium contains more components such as Staley’s vitamins (see medium DSZM 600) and Hunter’s salts (see medium DSZM 590), which includes 99 mg/L FeSO_4_. These observations are consistent with the fact that many planctomycetes grow better in nutrient-poor (oligotrophic) medium (Staley, 1973; Schlesner, 1994). In addition, not all Staley vitamins are needed for optimal growth, as noted in a previous study (Mishek et al., 2018). To better understand the mechanism associated with the improvement in growth obtained with the addition of *E. coli* culture filtrate, iron-free Caulobacter medium (which contains less nutrients than Staley’s medium) was retained as the baseline for further study on iron acquisition by *Gemmata* spp. Indeed, this study was suggested to us by the marked diversity of Planctomycetes lineages, including *Gemmata*-Isosphaera, Planctomyces, Phycisphaerae, Pirellula-Rhodopirellula-Blastopirellula and the “OM190” lineage, detected in iron-hydroxide deposits in association with other bacteria that synthetize bacterioferritin, which captures and stores ferric iron. The high diversity of Planctomycetes in these microbial-rich environments contrasts with the restricted diversity of Planctomycetes in some other environments, which suggests the existence of an iron-based cooperation between ordinary bacteria such as Proteobacteria (*E. coli* live in the human gut in association with *Gemmata* spp., Cayrou et al., 2013) and members of Planctomycetes (van Niftrik and Jetten, 2012; Storesund and Øvreås, 2013). Consistent with this hypothesis agar plate assays revealed that the *Gemmata* colonies near *E. coli* colonies are larger than those farther from *E. coli* colonies, which suggests the diffusion of unknown molecules that serve as potential growth factors for *Gemmata* spp. (Figure 3). In addition, the impregnation of FeSO_4_ at concentrations ranging from 10^–4^ to 10^–3^ M in blotting paper or solid agar plates resulted in rapid *Gemmata* spp. growth around the nitrocellulose disks, which was detected on days 4 and 5 (Figure 4A), whereas small colonies did not begin to appear until days 8 and 9 in more distant areas of the disk (Figure 4B). This effect was observed with both *Gemmata massiliana* and *Gemmata obscuriglobus*, even though a more dramatic effect was obtained with *Gemmata massiliana*. This finding suggests that both species can use iron under aerobic conditions. FeSO_4_ at a concentration ranging from 10^–4^ to 10^–3^ M promotes greater *Gemmata* spp. growth than FeCl_3_ at the same concentration; however, 0.2 to 10^–1^ M FeCl_3_ and 0.2 to 10^–1^ M iron is toxic for both species. The finding that deferoxamine slows down and prevents the growth of *Gemmata* spp. suggests that iron improves *Gemmata* spp. growth, as indicated in Figures 5, 6.

**FIGURE 7:**
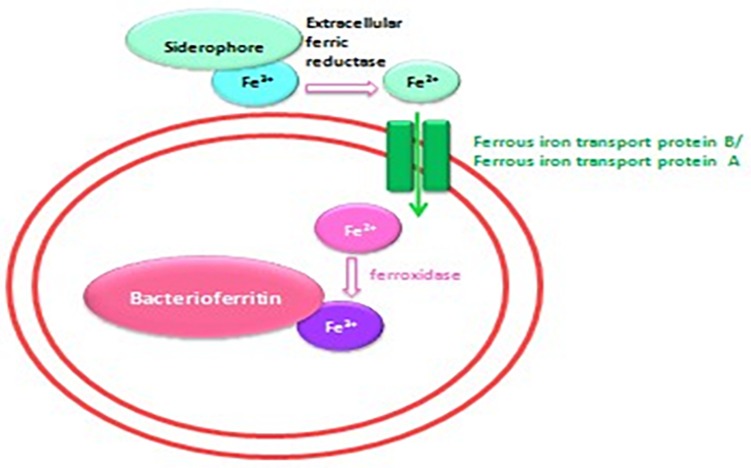
Schematic representation of the iron transport mechanism in bacteria.

Iron is a trace metal involved in many crucial biological processes as components of metalloproteins and serves as a cofactor or structural element for enzymes needed for bacterial survival and growth (Schalk et al., 2011). Iron found in soil, sediments and, more rarely, ocean water (Andrews et al., 2003) is extracted from the environment and transported into a bacterial cell by siderophores, which are repressed in an iron-rich environment. Additionally, environmental ferric iron must be reduced into ferrous iron by extracellular bacterial reductase for assimilation by bacteria (Vartivarian and Cowart, 1999; Guan et al., 2001; Miethke and Marahiel, 2007; D’Onofrio et al., 2010). The ferric uptake regulator protein controls iron acquisition through the ferrous iron-mediated repression of iron-regulated promoters because an excess of intracellular iron induces the production of reactive oxygen species via the Fenton reaction (Escolar et al., 1999). Therefore, several bacteria lacking siderophores depend on other bacteria to provide them with iron (Reeves et al., 1983; Posey and Gherardini, 2000; D’Onofrio et al., 2010), which partly explains the fastidiousness of these bacteria when grown on a synthetic medium (D’Onofrio et al., 2010). Accordingly, our observations revealed that the complementation of Caulobacter medium with *E. coli* culture filtrate and 10^–4^ M FeSO_4_ exerted a high growth-enhancement effect (*G. obscuriglobus*, 189 ± 22 colonies on day 1 and 1,091 ± 53 colonies on day 7; *G. massiliana*, 248 ± 19 colonies on day 1 and 1,029 ± 32 colonies on day 7) compared with that obtained with Caulobacter medium supplemented with *E. coli* filtrate alone (*G. obscuriglobus*, 134 ± 17 colonies on day 1 and 783 ± 31 colonies on day 7, *p* < 0.0016; *G. massiliana*, 166 ± 18 colonies on day 1 and 713 ± 27 colonies on day 7, *p* < 0.0122) (Figures 5, 6). The intracellular iron concentrations in *G. obscuriglobus* and *G. massiliana* cultured in an iron-depleted broth supplemented with *E. coli* filtrate were 0.63 ± 0.16 μmol/L and 0.78 ± 0.12 μmol/L, respectively, whereas concentrations of 1.72 ± 0.13 and 1.56 ± 0.11 μmol/L were found in *G. obscuriglobus* and *G. massiliana* grown in broth supplemented with *E. coli* filtrate and FeSO_4_. Under the other culture conditions, the iron concentrations in *G. obscuriglobus* and *G. massiliana* were 0.66 ± 0.17 and 0.52 ± 0.14 μmol/L, respectively. Hence, the addition of *E. coli* culture filtrate was found to act as a growth-promoting factor, and this finding raises questions regarding the nature of unknown growth-promoting factors in *E. coli* culture filtrate that improve the iron metabolism in microbial communities (D’Onofrio et al., 2010). In contrast, some siderophores produced by certain bacteria, such as deferoxamine by *Streptomyces*, could slow down and inhibit the growth of *Gemmata* and lead to the inability to isolate these bacteria via chelating iron. As indicated in Figures 5, 6, *E. coli* culture filtrate might contain siderophores that have higher affinity for iron than deferoxamine, which suggests that *E. coli* siderophores are able to shift the balance between deferoxamine and iron and make iron more available for cell growth. Additionally, our experiments revealed the aerobic oxidation of ferrous iron (the color of the Caulobacter liquid medium turned from light yellow to a color similar to that of iron rust after the addition of 7.5 μL of iron (to obtain a concentration of 0.2 M) at neutral pH (7.24), and it is possible that *E. coli* filtrate contains certain molecules, such as the ferrireductase enzyme, that can reduce ferric iron to promote iron uptake, as shown in Figure 7. The pH and ORP measured for all the media over the 7-day experiment ranged from 7 to 6, which suggests that the predominant form of iron is ferric iron (Supplementary Figures S1, S2). Both species can adapt to various culture conditions, including iron-replete and iron-depleted conditions, and regulate the pH under neutral conditions.

The analysis of the features of the colonies on solid Caulobacter agar complemented or not completed with *E. coli* filtrate (500 μL of solution A and 500 μL of solution B), FeSO_4_, FeCl_3_ and deferoxamine showed that the colonies grown on iron-enriched Caulobacter broth were bigger and redder in color than the colonies grown under the other culture conditions, which were small and pale pink in color. Although this phenomenon was observed with both *G. obscuriglobus* and *G. massiliana*, the effect on *G. obscuriglobus* was more dramatic, and the growth times to achieve visible colony formation in the presence of *E. coli* filtrate and FeSO_4_ (5–7 days for *G. obscuriglobus* and 6–7 days for *G. massiliana*) were shorter than those in media supplemented by FeSO_4_ or FeCl_3_ without *E. coli* filtrate (8–9 days for *G. massiliana*). Additionally, the bacteria showed moderate growth on Caulobacter solid agar after preincubation in broth containing deferoxamine (Figures 5, 6). Moreover, a slight growth-enhancement effect was observed in medium supplemented with *E. coli* filtrate and 10^–4^ M FeCl_3_, which might suggest that the presence of Cl slowed *Gemmata* growth compared with the presence of sulfate in FeSO_4_ because planctomycetes possess many sulfatases.

These data suggest that in the environment, as well as in human microbiota, *Gemmata* organisms might rely on neighboring bacteria to obtain the required amount of ferrous iron. In contrast, axenic media limit the ability of *Gemmata* bacteria to acquire iron because ferrous iron oxidizes into ferric iron at pH higher than 5, which results in a very low amount of available ferrous iron in axenic media. However, the growth-enhancing effect of filtrate and iron supplementation on the two species might only be explained by a potentiation mechanism. These results are encouraging, but further studies are needed to identify the potential growth factors secreted by *E. coli* via their purification and freeze-drying and to thus define approaches for enriching planctomycetes culture media.

In conclusion, our results indicate that not only ferrous iron but also *E. coli* culture filtrate, as a source of unknown growth factors that promote the rapid growth of *Gemmata* species, enhance *Gemmata* growth and can thus be used to improve the empirical culture media for Planctomycetes, as illustrated for *Gemmata* species in this study. This strategy involving the design of specific culture media helped improve the culture of fastidious bacteria and allows researchers to design specialized media from an empirical medium. Similarly, future investigation of the nutrients required by *Gemmata* organisms might aid the design of new culture media for their recovery from both environmental samples and host microbiota (Drancourt et al., 2014).

## Data Availability Statement

The raw data supporting the conclusions of this manuscript will be made available by the authors, without undue reservation, to any qualified researcher.

## Author Contributions

OK and RA performed the experiments and drafted the manuscript. SG and MD interpreted the data and drafted the manuscript.

## Conflict of Interest

The authors declare that the research was conducted in the absence of any commercial or financial relationships that could be construed as a potential conflict of interest.

## Footnotes

1 http://www.dsmz.de

## Supplementary Material

FIGURE S1 Daily oxidoreduction potential (ORP) of *Gemmata obscuriglobus*. The *Y* axis represents the ORP value, and the *X* axis represents the day of measurement.

FIGURE S2 Daily oxidoreduction potential (ORP) of *Gemmata massiliana*. The *Y* axis represents the ORP value, and the *X* axis shows the day of measurement.

## References

[B1] Aghnatios R. Cayrou C. Garibal M. Robert C. Azza S. Raoult D. (2015). Draft genome of *Gemmata* massiliana sp. nov, a water-borne Planctomycetes species exhibiting two variants. *Stand. Genomic Sci.* 10:120. 10.1186/s40793-015-0103-0 26649148 PMC4672568

[B2] Aghnatios R. Drancourt M. (2016). *Gemmata* species: Planctomycetes of medical interest. *Future Microbiol.* 11 659–667. 10.2217/fmb-2015-11 27158864

[B3] Andrews S. C. Robinson A. K. Rodríguez-Quiñones F. (2003). Bacterial iron homeostasis. *FEMS Microbiol. Rev.* 27 215–237. 10.1016/s0168-6445(03)00055-x 12829269

[B4] Bernander R. Ettema T. J. (2010). FtsZ-less cell division in archaea and bacteria. *Curr. Opin. Microbiol.* 13 747–752. 10.1016/j.mib.2010.10.005 21050804

[B5] Boedeker C. Schüler M. Reintjes G. Jeske O. van Teeseling M. C. F. Jogler M. (2017). Determining the bacterial cell biology of Planctomycetes. *Nat. Commun.* 8:14853. 10.1038/ncomms14853 28393831 PMC5394234

[B6] Cayrou C. Raoult D. Drancourt M. (2010a). Broad-spectrum antibiotic resistance of Planctomycetes organisms determined by Etest. *J. Antimicrob. Chemother.* 65 2119–2122. 10.1093/jac/dkq290 20699245

[B7] Cayrou C. Raoult D. Drancourt M. (2010b). Matrix-assisted laser desorption/ionization time-of-flight mass spectrometry for the identification of environmental organisms: the Planctomycetes paradigm. *Environ. Microbiol. Rep.* 2 752–760. 10.1111/j.1758-2229.2010.00176.x 23766281

[B8] Cayrou C. Sambe B. Armougom F. Raoult D. Drancourt M. (2013). Molecular diversity of the Planctomycetes in the human gut microbiota in France and Senegal. *APMIS* 121 1082–1090. 10.1111/apm.12087 23594317

[B9] Christen J.-R. Edmond E. Drancourt M. (2018). Methods for detecting *Gemmata* spp. bacteremia in the microbiology laboratory. *BMC Res. Notes* 11:11. 10.1186/s13104-017-3119-2 29310696 PMC5759251

[B10] D’Onofrio A. Crawford J. M. Stewart E. J. Witt K. Gavrish E. Epstein S. (2010). Siderophores from neighboring organisms promote the growth of uncultured bacteria. *Chem. Biol.* 17 254–264. 10.1016/j.chembiol.2010.02.010 20338517 PMC2895992

[B11] Drancourt M. Prebet T. Aghnatios R. Edouard S. Cayrou C. Henry M. (2014). Planctomycetes DNA in febrile aplastic patients with leukemia, rash, diarrhea, and micronodular pneumonia: FIG 1. *J. Clin. Microbiol.* 52 3453–3455. 10.1128/JCM.01207-14 24920769 PMC4313204

[B12] Escolar L. Pérez-Martín J. de Lorenzo V. (1999). Opening the iron box: transcriptional metalloregulation by the Fur protein. *J. Bacteriol.* 181 6223–6229. 10515908 10.1128/jb.181.20.6223-6229.1999 PMC103753

[B13] Franzmann P. D. Skerman V. B. D. (1984). *Gemmata obscuriglobus*, a new genus and species of the budding bacteria. *Antonie Van Leeuwenhoek* 50 261–268. 10.1007/BF02342136 6486770

[B14] Fuerst J. A. (2004). Planctomycetes: a phylum of emerging interest for microbial evolution and ecology. *WFCC Newslett.* 38 1–11.

[B15] Godinho O. Calisto R. Øvreås L. Quinteira S. Lage O. M. (2019). Antibiotic susceptibility of marine Planctomycetes. *Antonie Van Leeuwenhoek* 112 1273–1280. 10.1007/s10482-019-01259-7 30919144

[B16] Guan L. L. Kanoh K. Kamino K. (2001). Effect of exogenous siderophores on iron uptake activity of marine bacteria under iron-limited conditions. *Appl. Environ. Microbiol.* 67 1710–1717. 10.1128/aem.67.4.1710-1717.2001 11282625 PMC92789

[B17] Gudde L. R. Hulce M. Largen A. H. Franke J. D. (2019). Sterol synthesis is essential for viability in the Planctomycete bacterium *Gemmata obscuriglobus*. *FEMS Microbiol. Lett.* 366:fnz019. 10.1093/femsle/fnz019 30715321

[B18] Kaeberlein T. Lewis K. Epstein S. S. (2002). Isolating “uncultivable” microorganisms in pure culture in a simulated natural environment. *Science* 296 1127–1129. 10.1126/science.1070633 12004133

[B19] Kwon Y.-C. Jewett M. C. (2015). High-throughput preparation methods of crude extract for robust cell-free protein synthesis. *Sci. Rep.* 5:8663. 10.1038/srep08663 25727242 PMC4345344

[B20] Lage O. M. Bondoso J. (2012). Bringing Planctomycetes into pure culture. *Front. Microbiol.* 3:405. 10.3389/fmicb.2012.00405 23335915 PMC3538630

[B21] Lee K.-C. Webb R. I. Fuerst J. A. (2009). The cell cycle of the planctomycete *Gemmata obscuriglobus* with respect to cell compartmentalization. *BMC Cell Biol.* 10:4. 10.1186/1471-2121-10-4 19144151 PMC2656463

[B22] Lonhienne T. G. Sagulenko E. Webb R. I. Lee K.-C. Franke J. Devos D. P. (2010). Endocytosis-like protein uptake in the bacterium *Gemmata obscuriglobus*. *Proc. Natl. Acad. Sci. U.S.A.* 107 12883–12888. 10.1073/pnas.1001085107 20566852 PMC2919973

[B23] Meyer F. Paarmann D. D’Souza M. Olson R. Glass E. Kubal M. (2008). The metagenomics RAST server – a public resource for the automatic phylogenetic and functional analysis of metagenomes. *BMC Bioinformatics* 9:386. 10.1186/1471-2105-9-386 18803844 PMC2563014

[B24] Miethke M. Marahiel M. A. (2007). Siderophore-based iron acquisition and pathogen control. *Microbiol. Mol. Biol. Rev.* 71 413–451. 10.1128/mmbr.00012-07 17804665 PMC2168645

[B25] Mishek H. P. Stock S. A. Florick J. D. E. Blomberg W. R. Franke J. D. (2018). Development of a chemically-defined minimal medium for studies on growth and protein uptake of *Gemmata obscuriglobus*. *J. Microbiol. Methods* 145 40–46. 10.1016/j.mimet.2017.12.010 29292201

[B26] Pearson A. Budin M. Brocks J. J. (2003). Phylogenetic and biochemical evidence for sterol synthesis in the bacterium *Gemmata obscuriglobus*. *Proc. Natl. Acad. Sci. U.S.A.* 100 15352–15357. 10.1073/pnas.2536559100 14660793 PMC307571

[B27] Posey J. E. Gherardini F. C. (2000). Lack of a role for iron in the Lyme disease pathogen. *Science* 5471 1651–1653. 10.1126/science.288.5471.1651 10834845

[B28] Reeves M. W. Pine L. Neilands J. B. Balows A. (1983). Absence of siderophore activity in *Legionella* species grown in iron-deficient media. *J. Bacteriol.* 154 324–329. 6219988 10.1128/jb.154.1.324-329.1983 PMC217462

[B29] Riemer J. Hoepken H. H. Czerwinska H. Robinson S. R. Dringen R. (2004). Colorimetric ferrozine-based assay for the quantitation of iron in cultured cells. *Anal. Biochem.* 331 370–375. 10.1016/j.ab.2004.03.049 15265744

[B30] Sagulenko E. Morgan G. P. Webb R. I. Yee B. Lee K.-C. Fuerst J. A. (2014). Structural studies of Planctomycete *Gemmata obscuriglobus* support cell compartmentalisation in a bacterium. *PLoS One* 9:e91344. 10.1371/journal.pone.0091344 24632833 PMC3954628

[B31] Santarella-Mellwig R. Pruggnaller S. Roos N. Mattaj I. W. Devos D. P. (2013). Three-dimensional reconstruction of bacteria with a complex endomembrane system. *PLoS Biol.* 11:e1001565. 10.1371/journal.pbio.1001565 23700385 PMC3660258

[B32] Schalk I. J. Hannauer M. Braud A. (2011). New roles for bacterial siderophores in metal transport and tolerance. *Environ. Microbiol.* 13 2844–2854. 10.1111/j.1462-2920.2011.02556.x 21883800

[B33] Schlesner H. (1994). The development of media suitable for the microorganisms morphologically resembling Planctomyces spp., pirellula spp., and other Planctomycetales from various aquatic habitats using dilute media. *Syst. Appl. Microbiol.* 17 135–145. 10.1016/S0723-2020(11)80042-1

[B34] Schmidt J. M. Sharp W. P. Starr M. P. (1981). Manganese and iron encrustations and other features of Planctomyces crassus Hortobágyi 1965, morphotype Ib of the Blastocaulis-Planctomyces group of budding and appendaged bacteria, examined by electron microscopy and X-ray micro-analysis. *Curr. Microbiol.* 5 241–246. 10.1007/bf01571155

[B35] Seng P. Drancourt M. Gouriet F. La Scola B. Fournier P. Rolain J. M. (2009). Ongoing revolution in bacteriology: routine identification of bacteria by matrix-assisted laser desorption ionization time-of-flight mass spectrometry. *Clin. Infect. Dis.* 49 543–551. 10.1086/600885 19583519

[B36] Staley J. T. (1973). Budding bacteria of the pasteuria – blastobacter group. *Can. J. Microbiol.* 19 609–614. 10.1139/m73-100 4122691

[B37] Storesund J. E. Øvreås L. (2013). Diversity of Planctomycetes in iron-hydroxide 498 deposits from the Arctic Mid Ocean Ridge (AMOR) and description of *Bythopirellula* 499 goksoyri gen. nov., sp. nov., a novel Planctomycete from deep sea iron-hydroxide 500 deposits. *Antonie Van Leeuwenhoek* 104 569–584. 10.1007/s10482-013-0019-x 24018702

[B38] van Niftrik L. Devos D. P. (2017). Planctomycetes-verrucomicrobia-chlamydiae bacterial superphylum: new model organisms for evolutionary cell biology. *Front. Microbiol.* 8:1458 10.3389/fmicb.2017.01458 PMC5539593 28824586

[B39] van Niftrik L. Jetten M. S. (2012). Anaerobic ammonium-oxidizing bacteria: unique microorganisms with exceptional properties. *Microbiol. Mol. Biol. Rev.* 76 585–596. 10.1128/MMBR.05025-11 22933561 PMC3429623

[B40] Vartivarian S. E. Cowart R. E. (1999). Extracellular iron reductases: identification of a new class of enzymes by siderophore-producing microorganisms. *Arch. Biochem. Biophys.* 364 75–82. 10.1006/abbi.1999.1109 10087167

[B41] Vartoukian S. R. Palmer R. M. Wade W. G. (2010). Strategies for culture of ‘unculturable’ bacteria: culturing the unculturable. *FEMS Microbiol. Lett.* 309 1–7. 10.1111/j.1574-6968.2010.02000.x 20487025

[B42] Wagner M. Horn M. (2006). The Planctomycetes, Verrucomicrobia, Chlamydiae and sister phyla comprise a superphylum with biotechnological and medical relevance. *Curr. Opin. Biotechnol.* 17 241–249. 10.1016/j.copbio.2006.05.005 16704931

[B43] Winkelmann N. Harder J. (2009). An improved isolation method for attached-living Planctomycetes of the genus *Rhodopirellula*. *J. Microbiol. Methods* 77 276–284. 10.1016/j.mimet.2009.03.002 19303037

