# Supplementary material for: Escherichia coli Culture Filtrate Enhances the Growth of Gemmata spp
Source: Front Microbiol. 2019 Nov 6;10:2552. doi: 10.3389/fmicb.2019.02552 (PMC6851166; doi:10.3389/fmicb.2019.02552)
Supplement: FIGURE S1 — Daily oxidoreduction potential (ORP) of Gemmata obscuriglobus. The Y axis represents the ORP value, and the X axis represents the day of measurement. [file Presentation_1.PPTX]

## Slide 1
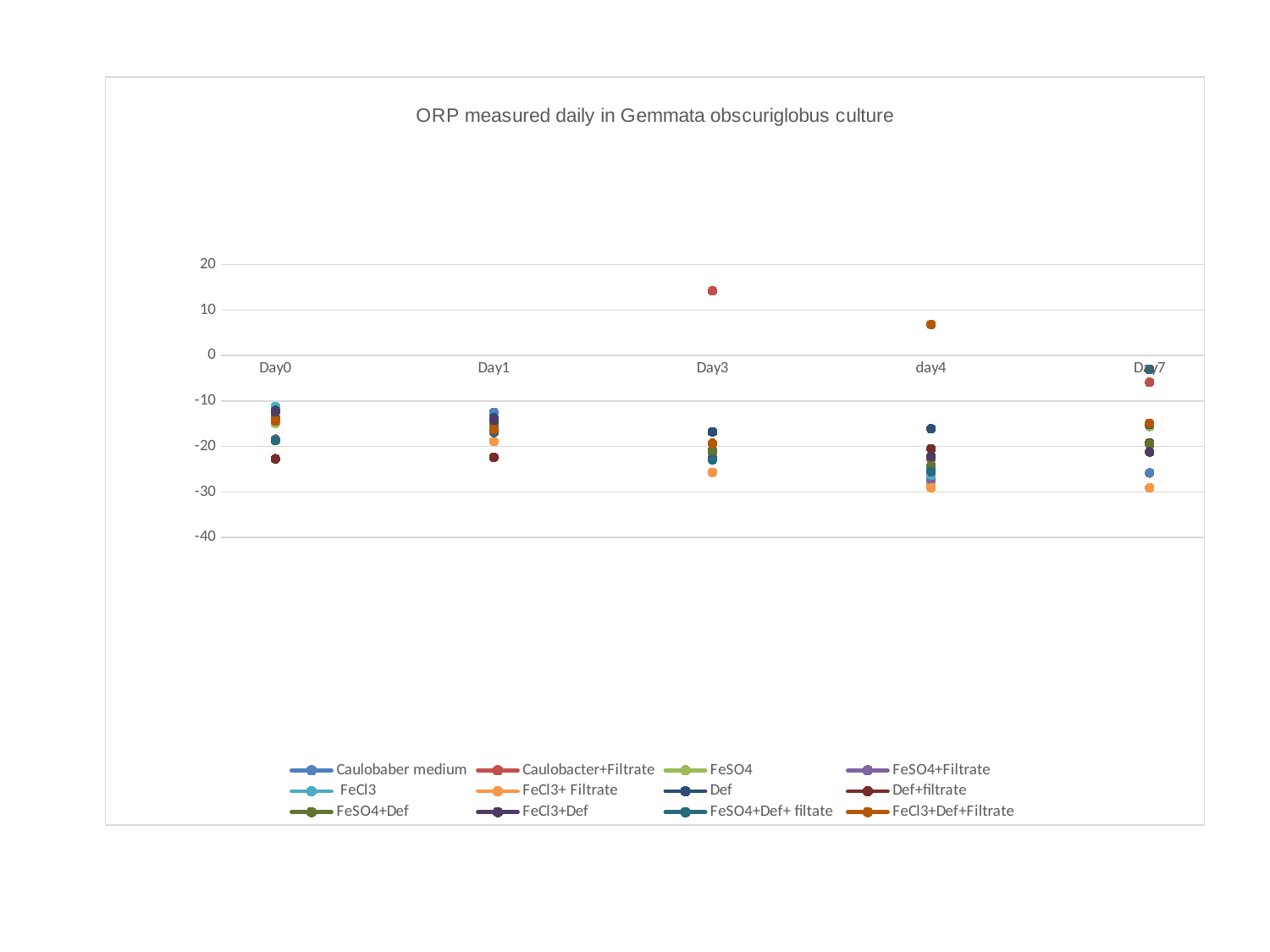

### Chart: ORP measured daily in Gemmata obscuriglobus culture
| Category | Caulobaber medium | Caulobacter+Filtrate | FeSO4 | FeSO4+Filtrate | FeCl3 | FeCl3+ Filtrate | Def | Def+filtrate | FeSO4+Def | FeCl3+Def | FeSO4+Def+ filtate | FeCl3+Def+Filtrate |
|---|---|---|---|---|---|---|---|---|---|---|---|---|
| Day0 | -13.7 | -14.5 | -14.9 | -18.4 | -11.2 | -18.7 | -12.1 | -22.7 | -13.7 | -12.4 | -18.7 | -14.4 |
| | None | None | None | None | None | None | None | None | None | None | None | None |
| Day1 | -12.5 | -14.6 | -14.0 | -15.6 | -13.8 | -18.9 | -13.7 | -22.4 | -15.9 | -14.3 | -16.9 | -16.3 |
| | None | None | None | None | None | None | None | None | None | None | None | None |
| Day3 | -22.5 | 14.2 | -22.3 | -21.2 | -20.8 | -25.7 | -16.8 | -22.7 | -20.9 | -19.3 | -22.9 | -19.3 |
| | None | None | None | None | None | None | None | None | None | None | None | None |
| day4 | -25.1 | -22.7 | -28.3 | -27.3 | -26.3 | -29.1 | -16.1 | -20.5 | -24.2 | -22.2 | -25.5 | 6.8 |
| | None | None | None | None | None | None | None | None | None | None | None | None |
| Day7 | -25.8 | -5.9 | -15.6 | -19.2 | -15.2 | -29.1 | -15.2 | -19.2 | -19.5 | -21.2 | -3.1 | -14.9 |
