# Supplementary material for: Escherichia coli Culture Filtrate Enhances the Growth of Gemmata spp
Source: Front Microbiol. 2019 Nov 6;10:2552. doi: 10.3389/fmicb.2019.02552 (PMC6851166; doi:10.3389/fmicb.2019.02552)
Supplement: FIGURE S2 — Daily oxidoreduction potential (ORP) of Gemmata massiliana. The Y axis represents the ORP value, and the X axis shows the day of measurement. [file Presentation_2.PPTX]

## Slide 1
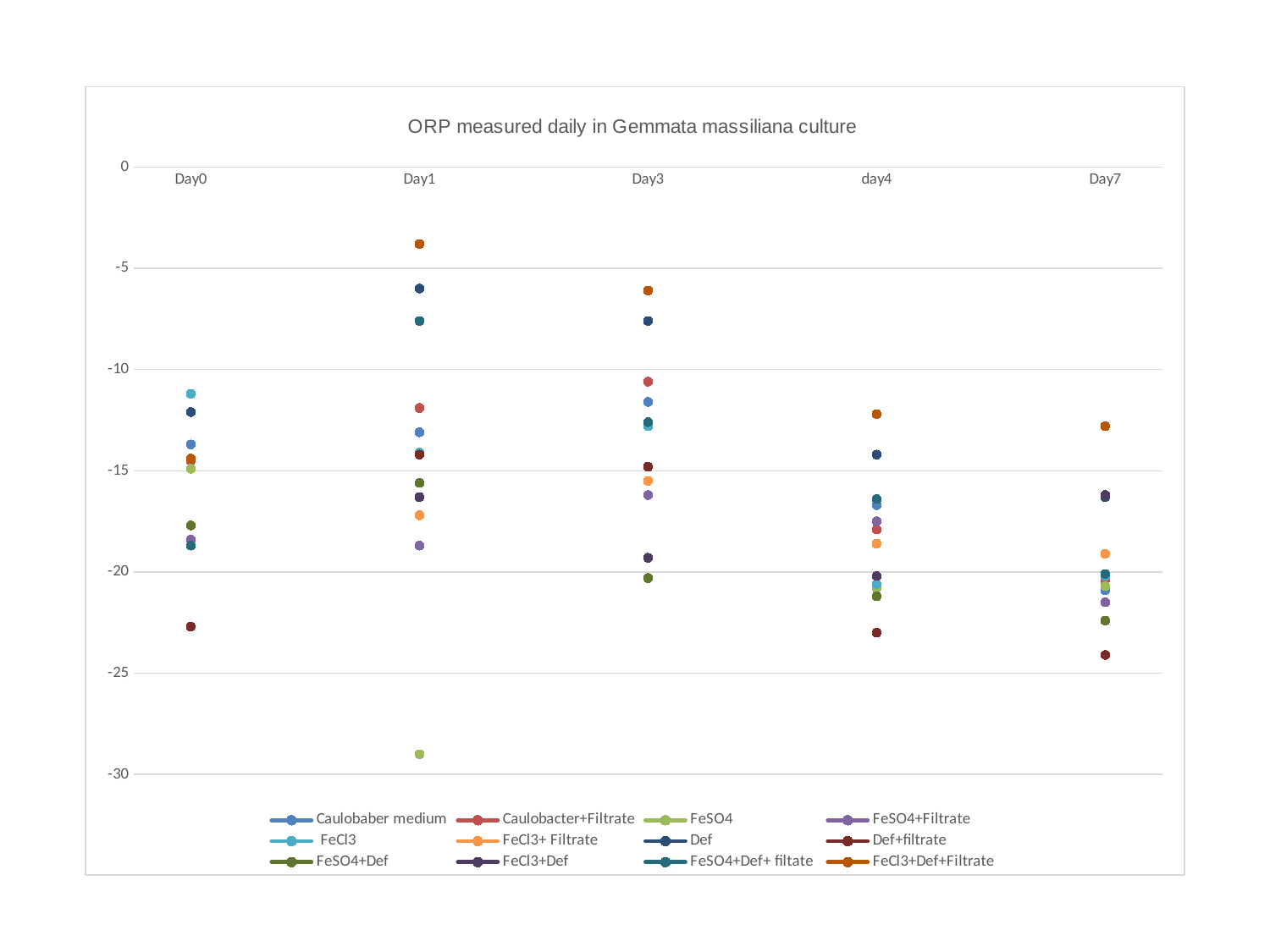

### Chart: ORP measured daily in Gemmata massiliana culture
| Category | Caulobaber medium | Caulobacter+Filtrate | FeSO4 | FeSO4+Filtrate | FeCl3 | FeCl3+ Filtrate | Def | Def+filtrate | FeSO4+Def | FeCl3+Def | FeSO4+Def+ filtate | FeCl3+Def+Filtrate |
|---|---|---|---|---|---|---|---|---|---|---|---|---|
| Day0 | -13.7 | -14.5 | -14.9 | -18.4 | -11.2 | -18.7 | -12.1 | -22.7 | -17.7 | -14.4 | -18.7 | -14.4 |
| | None | None | None | None | None | None | None | None | None | None | None | None |
| Day1 | -13.1 | -11.9 | -29.0 | -18.7 | -14.1 | -17.2 | -6.0 | -14.2 | -15.6 | -16.3 | -7.6 | -3.8 |
| | None | None | None | None | None | None | None | None | None | None | None | None |
| Day3 | -11.6 | -10.6 | -12.6 | -16.2 | -12.8 | -15.5 | -7.6 | -14.8 | -20.3 | -19.3 | -12.6 | -6.1 |
| | None | None | None | None | None | None | None | None | None | None | None | None |
| day4 | -16.7 | -17.9 | -20.8 | -17.5 | -20.6 | -18.6 | -14.2 | -23.0 | -21.2 | -20.2 | -16.4 | -12.2 |
| | None | None | None | None | None | None | None | None | None | None | None | None |
| Day7 | -20.9 | -20.4 | -20.7 | -21.5 | -20.2 | -19.1 | -16.3 | -24.1 | -22.4 | -16.2 | -20.1 | -12.8 |
